# Updates on Donor-Derived Infection in Solid Organ Transplantation, Report from the 2024 GTI (Infection and Transplantation Group) Annual Meeting

**DOI:** 10.3389/ti.2025.14237

**Published:** 2025-08-13

**Authors:** Carole Eldin, Paolo Antonio Grossi, Victoria Manda, Nassim Kamar, Olivier Lortholary, Hans H. Hirsch, Jean-Ralph Zahar, Vincent Michel Borderie, François Parquin, Eric Epailly, Florence Ader, Emmanuel Morelon, Edouard Forcade, David Lebeaux, Jérôme Dumortier, Filomena Conti, Agnes Lefort, Anne Scemla, Hannah Kaminski

**Affiliations:** ^1^ Maladies infectieuses et Tropicales - CHU Nord, Unité des Virus Emergents (UVE), (Aix-Marseille Univ, Università di Corsica, IRD 190, Inserm 1207, IRBA), Marseille, France; ^2^ Infectious and tropical Diseases Unit, Department of Medicine and Surgery- ASST-Sette Laghi, University of Insubria, Varese, Italy; ^3^ Department of Infectious Diseases, Hôpital Lariboisière, Saint-Louis-Lariboisière-Fernand Widal Hospitals, AP-HP, Paris, France; ^4^ Nephrology and Organ Transplantation Unit, Centre Hospitalo Universitaire Rangueil, INSERM U1043, Structure Fédérative de Recherche Bio-Médicale de Toulouse, Paul Sabatier University, Toulouse, France; ^5^ Institut Pasteur, Université Paris Cité, National Reference Center for Invasive Mycoses and Antifungals, Translational Mycology Research Group, Mycology Department, Paris, France; ^6^ Department of Biomedicine, Transplantation and Clinical Virology, University of Basel, Basel, Switzerland; ^7^ Département de Microbiologie Clinique, Centre Hospitalier Universitaire Avicenne, Assistance Publique - Hôpitaux de Paris, Bobigny, France; ^8^ Hôpital National des 15-20, Paris, France; ^9^ Service de Chirurgie Thoracique et Transplantation Pulmonaire, Hôpital Foch, Suresnes, France; ^10^ Department of Cardiology and Cardiovascular Surgery, Hôpitaux Universitaires de Strasbourg, Strasbourg, France; ^11^ Infectious Diseases Department, Croix-Rousse Hospital, Hospices Civils de Lyon, Lyon, France; ^12^ Department of Transplantation, Edouard Herriot University Hospital, Hospices Civils de Lyon, University Lyon, University of Lyon I, Lyon, France; ^13^ Service d’Hématologie Clinique et Thérapie Cellulaire, Centre Hospitalier Universitaire de Bordeaux, Hôpital Haut Lévêque, Bordeaux, France; ^14^ Service de Microbiologie, Unité Mobile d’Infectiologie, Assistance Publique - Hôpitaux de Paris, Hôpital Européen Georges Pompidou, Paris, France; ^15^ Hospices Civils de Lyon, Hôpital Edouard Herriot, Fédération des Spécialités Digestives, et Université Claude Bernard Lyon 1, Lyon, France; ^16^ Assistance Publique-Hôpitaux de Paris (Assistance Publique - Hôpitaux de Paris), Pitié-Salpêtrière Hospital, Department of Medical Liver Transplantation, Paris, France; ^17^ IAME Infection Antimicrobials Modelling Evolution, UMR1137, Université Paris-Cité, Paris, France; ^18^ Department of Internal Medicine, Beaujon University Hospital, Assistance Publique - Hôpitaux de Paris, Paris, France; ^19^ Department of Nephrology and Kidney Transplantation, Necker-Enfants Malades Hospital, Assistance Publique-Hôpitaux de Paris, Paris, France; ^20^ Department of Nephrology, Transplantation, Dialysis and Apheresis, Bordeaux University Hospital, Bordeaux, France

**Keywords:** donor-derived infection, chronic hepatitis, Bk virus, HIV, non-candida derived fungal infection

## Abstract

The annual meeting of the French GTI (Transplantation and Infection Group) focused on donor-derived infections (DDIs) in solid organ transplant (SOT) recipients. Given the ongoing organ shortage, rigorous donor screening is essential to detect potential infectious risks. Donor evaluation should include medical history, travel, vaccination status, serologies, and exposures. Various pathogens are of concern, including viruses (HIV, hepatitis, BK polyomavirus), multidrug-resistant bacteria, fungi, and emerging arboviruses like West Nile virus and dengue. HIV-positive donor to HIV-positive recipient (D+/R+) transplantations are increasingly accepted, with promising outcomes. Hepatitis E (HEV) is now the most common viral hepatitis and may lead to chronic infection in SOT recipients, requiring ribavirin treatment. Non-Candida fungal infections, though rare, are associated with high mortality and demand early recognition. Climate change and globalization are expanding the range of vector-borne infections, necessitating seasonal and regional screening. BK polyomavirus remains a major complication in kidney transplant recipients, and monitoring viral load is critical. Bacterial infections from donors are uncommon but should be evaluated based on site, organism, resistance profile, and treatment history. Overall, maintaining safety in transplantation requires constant vigilance, updated knowledge, and personalized risk-benefit analysis to adapt to emerging infectious threats—especially amid ongoing organ scarcity.

## Introduction

The French annual meeting of the GTI (“Groupe Transplantation et Infection”) focused this year on donor-derived infections in solid organ transplant recipients. We summarize in this report the presentations and discussions around the covered topics, highlighting current challenges and expert opinions. Each topic includes new insights/perspectives on clinical presentation, diagnostic and prevention strategies, and risk management protocols for those at-risk for donor-derived infections.

## Overview of Donor-Derived Infections

In Europe in 2022, 27,952 organ transplants were performed, but over 40,000 patients were on a waiting list. The challenge is to strike a balance between availability and need, as transplant candidates may die while waiting, and donor-derived infections are possible complications of solid organ transplantation (SOT). The 8th edition of the EDQM Guide on Quality and Safety of Organs for Transplantation has been published in 2022 and is available online^1^ [1]. To mitigate the infectious risk, it is suggested to collect [2]: medical history including prior infections, vaccinations (with an emphasis on live attenuated vaccines), occupational exposures, travel history without limit time before transplantation, receipt of transfusions of blood or blood products, human immunodeficiency virus (HIV, hepatitis B virus (HBV), hepatitis C virus (HCV) serostatus or other transmissible diseases, tattooing, ear or body piercing, drug use, sexual behavior, jail incarceration, pets or zoonotic contacts. Basic screening for infections in deceased organ donors is summarized in [1]. Depending on specific risk factors identified in the donor, other tests may be required.

Blood HIV, HBV, HCV nucleic acid amplification tests may be used in the “window period,” when serology is still negative. Globalization (migration, tourism, global trade) is an important factor underlying the emergence and re-emergence of specific infectious diseases. The 8th edition of the Guide on the quality and safety of organs intended for transplantation has published a table of possible risks of transmissible infections according to geographical areas. These tests should be considered for screening symptomatic donors who have lived and/or traveled to one of several of these endemic areas and whom may have been exposed or at risk of vertically acquired infection. Additionally, receipt of live attenuated vaccine within 4 weeks prior to transplantation by the patient or his/her close contacts should be investigated because of the risk of reactivation or transmission upon immunosuppression.

Some specific guidelines are summarized below:

In cases of herpes simplex virus (HSV 1 or 2) serological mismatch, severe hepatitis have been reported, and given that transmission may occur with any organ, the Swiss guidelines advise that all transplant recipients with HSV serological mismatch should receive a 6-month course of antiviral prophylaxis [3].

Disseminated toxoplasmosis after transplantation is rare but can be fatal due to delayed diagnosis and treatment. The U.S. Organ Procurement and Transplantation Network now recommends screening of all deceased donors.

Donor-derived HTLV-1 is another challenge in solid organ transplantation, but the approach is heterogeneous across countries. In France, Spain, and the UK all donors are screened regardless of risk factors, while in other countries screening is limited to donors who have lived and/or traveled to endemic areas. In Europe, Romania is reported to be the only country with a high prevalence of HTLV-1.

Five arboviruses require our attention in view of their emergence in different parts of the world: yellow fever, dengue (DENV), Zika, West Nile (WNV), chikungunya, transmitted by blood and organs [4], and will discussed later in a specific chapter.

Romagnoli R, et al. [5] published the results of the first 10 liver transplants with proven SARS-CoV-2 positive donors, suggesting a very low risk of transmission with liver transplantation. Nevertheless, the comparison of the 2 populations of transplanted livers from COVID-19 donors with non-COVID 19 donors showed more frequent hepatic artery thrombosis, which would deserve to be explored in the future. Data are awaited on the risk associated with the use of organs (apart from lungs, which are systematically rejected) from donors with proven SARS-CoV-2 infection, with emphasis on the timing of the procedure in relation to the onset of immunological protection.

## Transplantation in HIV

Dr. Victoria Manda, from Saint-Louis/Lariboisière University Hospital, Paris, France, presented an overview of current practices worldwide regarding solid-organ transplantation in people living with HIV (PLWH).

As of 2022, the global population of PLWH was estimated at around 39 million, with 1.3 million new infections, mainly in South America and Africa [UNAIDS 2023]. The risk of chronic kidney disease is higher in PLWH compared to the general population, becoming a significant public health concern by accelerating disease progression and complicating treatment. Kidney transplantation (KT) is the standard treatment for end-stage renal disease [6], but access remains limited for PLWH compared to HIV-negative patients.

The first KT from an HIV-positive deceased donor (D) to an HIV-positive recipient (R) was performed in South Africa in 2010. The first liver transplant from an HIV-positive living donor to an HIV-positive recipient took place in the United States in 2017 [7]. In South Africa, between 2008 and 2014, 27 PLWH received KT. All recipients had CD4^+^ T-cell counts above 200/mm^3^ and were on highly active antiretroviral therapy (HAART) with undetectable HIV viral loads. Some donors had not previously received ART, while others had only received first-line combinations.

Patient survival rates were 84%, 84%, and 74% at 1, 3, and 5 years post-transplant, respectively. Graft survival rates were 93%, 84%, and 84% over the same time periods. Overall rejection rates were 8% at 1 year and 22% at 3 years. HIV viral loads remained undetectable post-transplantation [8].

Following the passage of the HIV Organ Policy Equity (HOPE) Act in 2013, HIV D+/R+ transplants can be performed under research protocols in the U.S. To inform policy and practice—especially regarding whether this approach should continue—multicenter pilot studies were conducted to assess the feasibility and safety of liver or kidney transplantation involving HIV-positive donors (D+/R+) versus HIV-negative donors to HIV-positive recipients (D-/R+) [9, 10]. Donor selection required no current or prior opportunistic infections, recipient use of HAART, and favorable results from a donor pre-implantation biopsy.

A prospective multicenter pilot study specifically examined the safety and risks associated with HIV+ donors for KT by directly comparing HIV D+/R+ and HIV D-/R+ cases [9]. From 2016 to 2019, across 14 centers, 75 HIV+ kidney transplants were performed (25 D+ and 50 D-), with a median follow-up of 1.7 years. There were no deaths or differences in 1-year graft survival, estimated glomerular filtration rate, HIV breakthrough, infectious hospitalizations, or opportunistic infections. However, delayed graft function occurred significantly more often in D+ cases than in D-. One-year rejection rates were also higher in D+ recipients but did not reach statistical significance. Lymphocyte-depleting induction therapy was associated with lower rejection rates. The authors noted that the trend toward higher rejection in D+ cases raised concerns and recommended further research [9].

Of note, passive HIV strain transfer from a viremic HIV-positive donor to an HAART-treated HIV-positive recipient was detected in blood and urine for up to 16 days post-transplantation, but not beyond [10].

In cases of liver failure, only a few case reports exist of HIV D+/R+ liver transplantation (LT), all with limited follow-up. Therefore, as with KT, a prospective multicenter pilot study was conducted comparing HIV D+/R+ LT to HIV D-/R+ LT [11]. Between 2016 and 2019, 45 LT recipients (including 8 simultaneous LT-KT cases) were enrolled across 9 centers. The cohort included 24 HIV D+/R+ and 21 HIV D-/R+ patients, with a median follow-up of 23 months. The median CD4 count was 287 cells/μL, and all recipients were on antiretroviral therapy. Additionally, 56% were hepatitis C virus (HCV) seropositive, and 13% were HCV-viremic.

One-year weighted survival was significantly higher in the D- group than in the D+ group (100% vs. 83%, p = 0.04). There were no significant differences in 1-year graft survival, rejection, HIV breakthrough, or serious adverse events. However, the D+/R+ group experienced more opportunistic infections, infection-related hospitalizations, and cancer cases, warranting further investigation [11].

In France in 2023, 325 PLWH were on the waiting list for KT and 20 for LT [12]. A program was launched in 2022 to evaluate HIV+ donors for PLWH candidates. For deceased donors, criteria are stringent: brain death must be confirmed, the donor must be on HAART with a viral load below 50 copies/mL for at least 1 year, and antiviral therapy must be stable. No opportunistic infections should be present at the time of donation. No minimum CD4 count is required. HIV proviral DNA genotyping is expected to be feasible through biocollection of the donor’s plasma. French guidelines do not specify antiviral resistance criteria, as an undetectable viral load is mandatory for donor eligibility.

For living donors, the decision is made on a case-by-case basis by an independent multidisciplinary expert committee. HIV-positive individuals with well-controlled infections and no comorbidities may be considered low-risk kidney donors. Recipient eligibility criteria are the same as those for HIV-negative organs. No data is currently available on HIV D+/R− transplantation.

## Donor-Transmitted Infections - Viral Hepatitis

Nassim Kamar, Toulouse University Hospital, France, presented an overview about donor-transmitted viral hepatitis.

### Hepatitis E Virus (HEV)

Currently, HEV infection is the most common viral hepatitis. HEV is an RNA virus that is still underdiagnosed. Genotypes 1 and 2 (G1 and G2) are strictly human, of orofecal transmission, G3 & 4 are a zoonosis. Chronic HEV is defined by replication beyond 3 months after infection.

In case of HEV infection in SOT, around two-thirds developed chronic hepatitis. The use of tacrolimus rather than cyclosporin A is one of the main predictive factors for chronic hepatitis [13]. Immunosuppressant dose reduction may be a first-line therapeutic option. The successful use of ribavirin (RBV) for the treatment of chronic HEV infection was described in 59 immunosuppressed recipients of SOT [14] at a median dose of 600 (29–1200) mg/day, and a median therapy duration 3 (1–18) months. The duration of therapy can be optimized according to HEV fecal shedding [15]. Indeed, before stopping ribavirin, HEV RNA should be negative both in the serum and the stools. In case, HEV RNA is not detected anymore in the serum but still detected in stools, ribavirin should be pursued and HEV RNA should looked for monthly until both become negative which allow stopping ribavirin.

In case of failure, PEGylated-interferon-α has been successfully used in liver-transplant patients, but it is contraindicated in other organ recipients because it increases the risk of acute rejection [16].

Human-to-human transmission of HEV 3&4 is possible but rare, through the blood or the organs. HEV RNA is detected in roughly 1 out of 1200 blood donors. Systematic screening has been implemented in the UK; the incidence of HEV in the organs of deceased donors is 0.94 per 1,000, which is approximately four times higher than in their blood [17].

**FIGURE 1:**
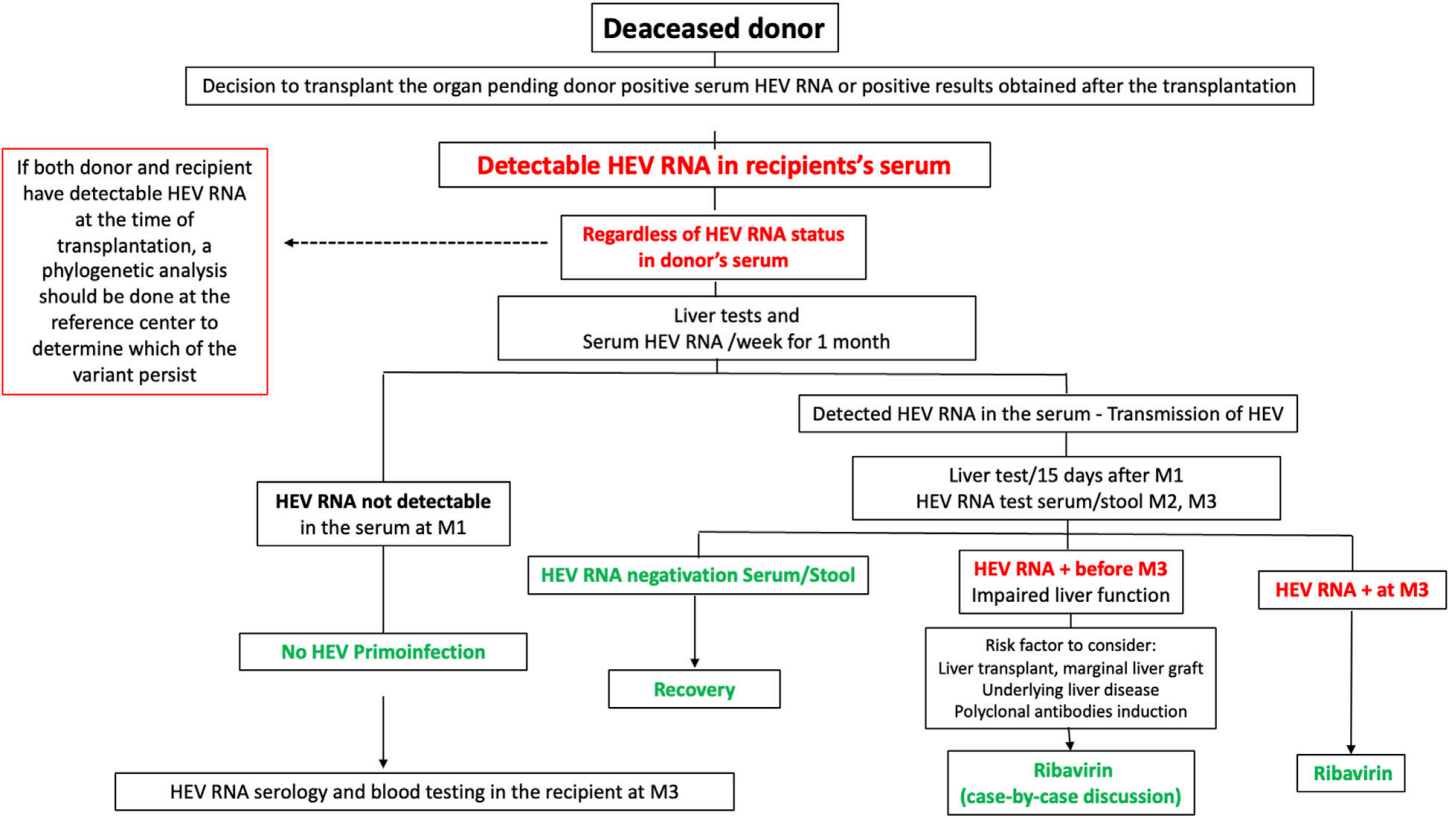
Recommendations in France for cases where HEV RNA is detected in the recipient’s blood following transplantation from a HEV-positive deceased organ donor.

In France, HEV RNA is now screened in donors’ sera. If HEV RNA is detected in a living donor, transplantation is delayed. In deceased donors, if this information is available before harvesting, the transplantation team should inform the recipient. Sometimes, donor HEV RNA positivity information may only become available after transplantation. In both cases, hepatic surveillance is recommended. If the recipient becomes positive for more than 1 month, a treatment by RBV is discussed individually, and if positive for 3 months, RBV is given. Figure 1 summarizes the recommendations in France for cases where HEV RNA is detected in the recipient’s blood following transplantation from a HEV-positive deceased organ donor.

### Hepatitis C (HCV)

The lastest KDIGO Guidelines recommend that all chronic renal disease (CRD) and KT patients with HCV infection are evaluated for direct-acting antiviral (DAA)-based therapy [18]. DAA therapy should be administered to all HCV-infected transplant candidates, either before or after transplantation. Factors guiding timing of HCV treatment (before vs. after kidney transplantation) include donor type (living vs. deceased donor), anticipated waiting-list time by donor type, severity of hepatic fibrosis, and willingness of the patient and program to accept an organ from an HCV-infected donor. The KT from HCV deceased donor into HCV recipients followed by early post-transplant treatment with DAA agents successfully shortened the waiting time for HCV-infected kidney transplant candidates [19].

In France, since 2023, the eligibility criteria for recipients have been expanded to include all organ recipients, regardless of whether the donor is HCV serology positive or negative, as long as the donor’s RNA is negative. The risk of HCV transmission from a donor with a positive anti-HCV antibody and negative viral genomic screening (VGM) is estimated to be between 1% and 9% in liver transplantation’.

A systematic PCR monitoring in post transplantation and DAA enable a 100% sustained virologic response.

### Hepatitis B (HBV)

In France since 2023, the transplantation of kidneys from deceased donors who are hepatitis B surface antigen (HBsAg)-positive and/or viremic (HBV DNA-positive) has been possible for recipients who are HBsAg-positive and/or viremic. Recipients must be treated in post transplantation with anti-HBV therapy during at least 6–12 months and longer as HBsAg or viremia remain positive.

The utilization of HBV NAT+ allografts into seronegative recipients is not authorized in France. It has been investigated in US [20]. KT and LT patient and allograft survival were not different between HBV NAT+ and HBV NAT- recipients whereas HBV NAT+ KT recipients had shortened waitlist time and pretransplant duration on dialysis.

## Donor Derived Non-Candida Fungal Infections in Solid Organ Transplant (SOT) Recipients

Olivier Lortholary, Necker University Hospital, France, then addressed donor derived non-*Candida* fungal infections.

Data from the Transplant-Associated Infection Surveillance Network (TRANSNET), a prospective study performed in 23 transplant centers throughout the U.S, demonstrated that the most frequent non-Candida invasive fungal infections (IFI) were invasive aspergillosis (19%), cryptococcosis (8%), non-*Aspergillus* molds (8%) and endemic fungi (5%) [21].

Donor derived fungal infections are very rare but may have a poor prognosis. Thus, an early unusual or severe infection after SOT should suggest graft transmitted IFI.

**FIGURE 2:**
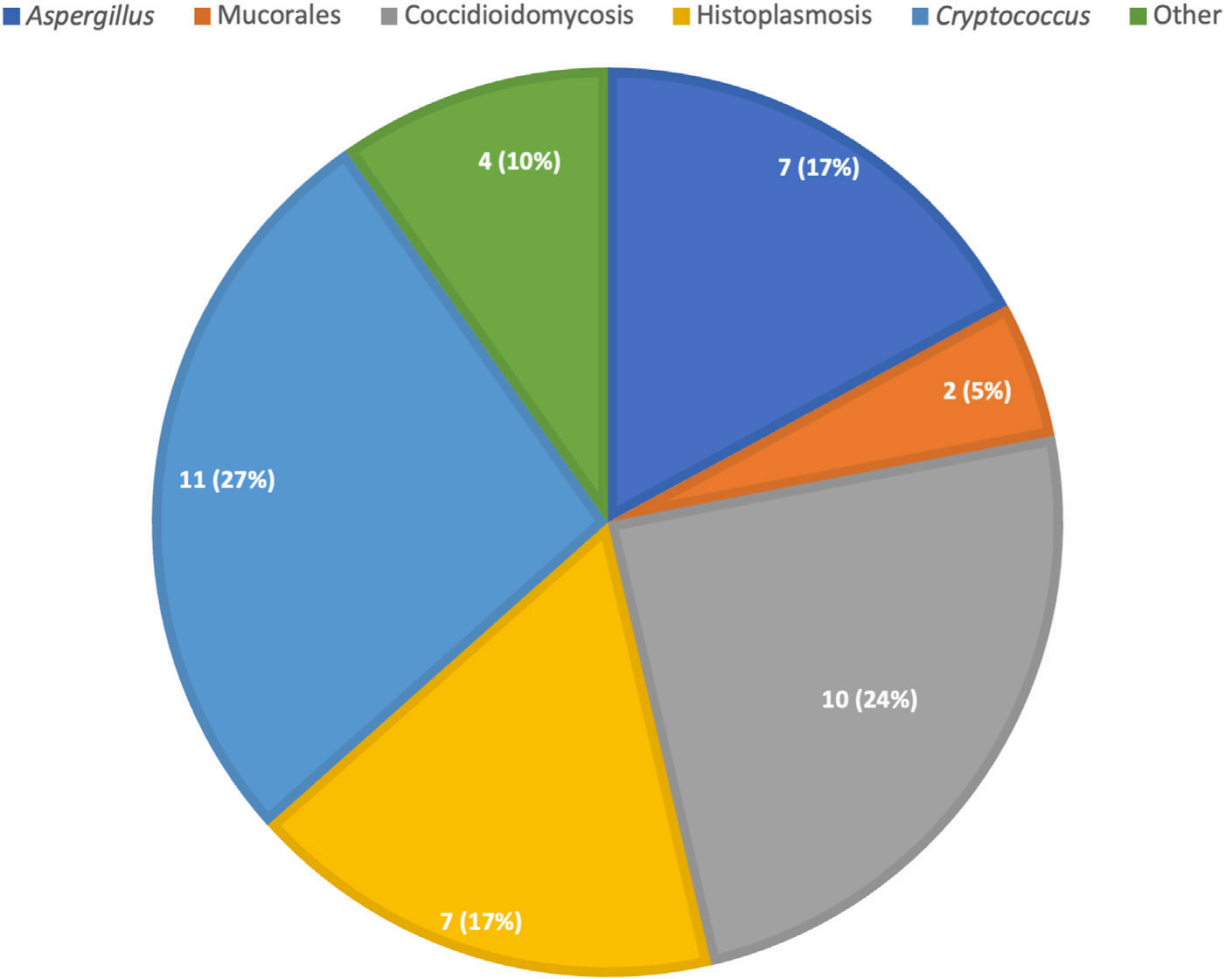
Distribution of proven/probable donor derived non-candida fungal infections, absolute count (percent).

In the 10 years analysis of donor-derived infections in the U.S, the mortality rate was 15% for all infections but it was considerably higher for certain fungi, particularly *Coccidioides* [22]. Delay in diagnosis likely contributed to the high mortality as these diseases may present with diffuse, difficult-to-recognize symptoms in the posttransplant period. In this study, coccidioidomycosis, aspergillosis, cryptococcosis and histoplasmosis were the most frequent non-*Candida* IFI. The distribution of proven/probable donor-derived non-*Candida* fungal infection is recapitulated in Figure 2. As the number of donors coming from endemic areas increases, travel to endemic areas should be investigated, when possible, in donors.

### Donors With Active or Latent Invasive Fungal Disease (IFD)

The circumstance is rare but potentially severe for graft and or recipient. The graft can be contaminated by an infected donor or by an organ contaminated at the time of sampling/transportation (for example, the presence of *candida* in the preservation fluid). The criteria for classification of donor-derived fungal infections were defined in 2012 [23].

To avoid such complications, sampling is contraindicated when donors are known to have an active fungal infection. However, donor active infections may be unknown at time of transplantation: in potential donors with unexplained meningoencephalitis, cryptococcosis screening should be considered. Descriptions of donor-derived *cryptococcosis* also showed reactivation of a latent infection in the transplanted organ [24]. Mold infections as *Aspergillus fumigatus and Scedosporium apiospermum* have also been acquired through unrecognized infections in the donor. Transplant tourism represents another concern: IFI, frequently originating at the graft site have emerged as a devastating complication and are associated with high rates of graft loss and death [25].

Regarding endemic mycoses, few cases have been described [26].

A prospective study performed in 15 transplant centers throughout the U.S, found only 33 cases of dimorphic mycoses among 16,806 patients who received a SOT during the 5-year study period; 23 histoplasmosis, 6 coccidioidomycosis, and 4 blastomycosis, most of them were primary infections, not transmitted by the donor [27].

*Histoplasmosis* occurs in only 0.1%–0.5% of transplant recipients from endemic areas and 1%–5% of healthy subjects have positive tests for Histoplasma antigen or antibodies, primary infection being the dominant mode of acquisition. When disease is transmitted via an infected allograft, the infection is most likely transmitted via the liver allograft. Itraconazole treatment, until antigen clearance is met, is recommended for the management of histoplasmosis in living donor. *Coccidioidomycosis* is another endemic mycosis, mainly in the southwest of United States, and may lead to vasculitis and meningoencephalitis in the recipient. Its first-line therapy in donors is fluconazole [28].

### The Contamination of Preservation Fluid

While *Candida* spp are from far the most frequent fungal source of contamination fluid, *Aspergillus* contamination has also been described [29].

Both infections may lead to devastating abscesses and arteritis, with high transplant removal and death rates. Thus a systematic fungal culture of preservation fluid could be discussed and is already recommended in France [30].

In the future, increasing rates of travels and migrations, as well as changing climate, may increase specific risks for transplant recipients, especially those of transmitted or primary acquired endemic mycoses.

## Emerging ArboViruses in Transplantation

Dr. Carole Eldin, infectious disease physician and member of the UVE research unit (Unité des Virus Emergents), Marseille, France, presented an update on emerging arboviruses in SOT settings. Climate change is associated with an expansion of the density and distribution area of many vectors (mainly mosquitoes) and an increase in vectorial capacity, leading to the emergence of arboviruses.

### West Nile Virus (WNV)

West Nile virus is an Orthoflavivirus transmitted by Culex mosquitoes from animals (mostly birds, which are the reservoir, or horses) to humans. The only way of human-to-human transmission is through SOT or blood transfusion. The incidence of WNV infection is increasing in Europe [31]. In France, a cluster was described in the South-West (Gironde) in 2023, where 32 cases were reported, including 4 blood donors.

WNV infections in solid organ transplant (SOT) recipients can occur following SOT from an infected donor or via a mosquito bite after SOT. Infections are more severe in SOT recipients and can lead to neuroinvasive disease, mainly encephalitis, in 40%–60% of infected patients [32, 33].

Prevention of the infection is based on the screening of organ donors (PCR and serology), which has been recommended by the French High Council of Public Health [34] since 2020 for donors who have been exposed to risk areas during the virus circulation period up to 28 days before organ donation. The list of at-risk areas is regularly updated [35]. The result of WNV screening should be known before transplantation, and the donation should be postponed in case of a positive result, particularly in the case of living donation. However, improving access to screening techniques is still in progress to allow for real-time decision-making.

**FIGURE 3:**
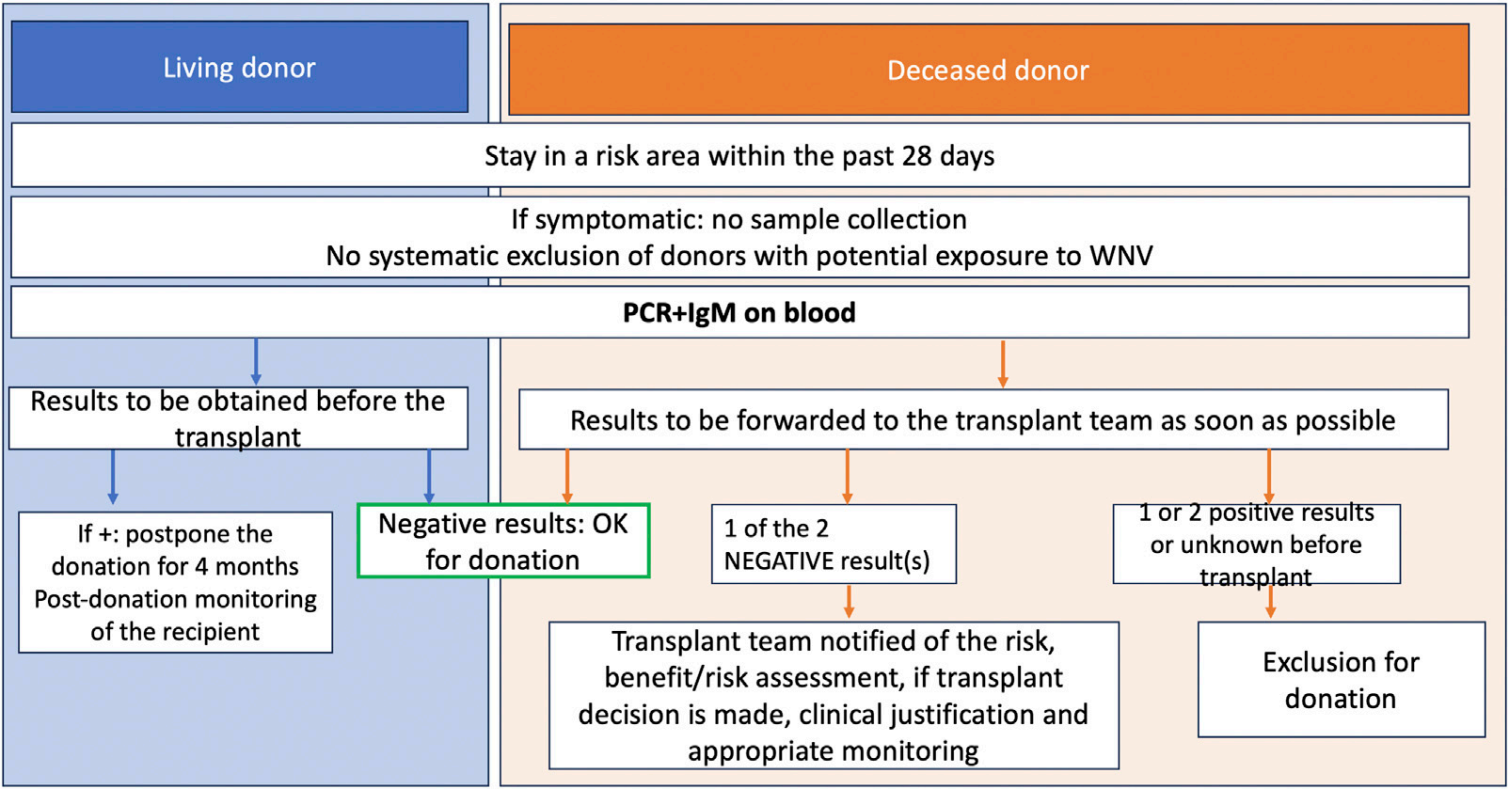
Biomedecine National Agency algorithm, donor screening for West Nile Virus.

In case of a diagnosis of infection in a SOT recipient, there are no established treatment guidelines, but some cases have reported the use of immunomodulation, intravenous immunoglobulins [36], interferon, or prophylaxis with ribavirin or WNV antibodies from convalescent plasma [37, 38]. We summarize the current French National Biomedicine agency in Figure 3.

### Tick-Borne Encephalitis (TBE)

This arbovirus is mainly transmitted to humans by an Ixodes tick bite.

Transmission of TBE from a deceased donor to three SOT recipients (two kidneys and one liver) has been reported in Poland [39], leading to death.

Since 2020, the HCSP has recommended the inclusion of a history of tick bites within the past 28 days in the organ pre-donation questionnaires. In case of a positive response in an at-risk geographical area and period, specific molecular and serological tests should be performed. No treatment is currently available.

### Dengue (DENV)

The world has seen a historic increase in cases of dengue in recent years [40]. In France, in 2022, 35 autochthonous cases were reported, and an autochthonous cluster of 3 cases in the Paris area confirmed the emergence of this virus [41]. Some cases of dengue after renal transplantation have been reported, notably in French overseas territories (Réunion Island) [42, 43]. The particularity of some cases is that the negative blood test results from donors were not sufficient to prevent transmission to the recipient. This report suggests that the kidney may be a potential viral reservoir for dengue virus. To date, there is no specific prevention measure in mainland France regarding transplantation.

## BK Polyomavirus–Focus on Kidney Transplantation

Hans H. Hirsch, University of Basel, Switzerland, presented recent data and a summary of 2024 international guidelines about BK polyomavirus in kidney transplants.

The BK polyomavirus (BKPyV) is a small, environmentally resilient virus with a circular, double-stranded DNA genome. While its natural transmission route remains unclear, it likely reaches the reno-urinary tract via the bloodstream, establishing lifelong persistence [44]. Approximately 10% of BKPyV-seropositive blood donors have low levels of the virus in their urine [45] highlighting the role of donor-derived BKPyV in kidney transplantation.

BKPyV persists in the reno-urinary tract through several mechanisms, including latency, reactivation within host cell nuclei (undetectable by the immune system until cell lysis), agnoprotein expression that disrupts innate immune sensing, and viral genome variations leading to serotypes and variants [46]. There are four main BKPyV serotypes: I (most common, 71% of cases), IV (19%), II (8%), and III (2%). Genetic and immunological differences between these serotypes influence post-transplant BKPyV-related risks [47].

In immunosuppressed patients, BKPyV is linked to three major complications: BKPyV nephropathy (BKPyVAN) in kidney transplantation; BKPyV hemorrhagic cystitis in hematopoietic cell transplantation; BKPyV-associated urothelial carcinoma, often years after poorly controlled BKPyVAN [48].

For kidney transplant patients, multiple prospective studies have confirmed the continuum of presentations [49]: no/low level viruria (<100,000 copies/mL) in 60%–80%; high-level viruria (decoy cells or >10 million copies/mL) in 20%–40%; the new-onset plasma BKPyV-DNAemia in 5%–21% after 2–6 weeks, and eventually the invasive diagnosis of biopsy-proven BKPyV-nephropathy initially without and then with declining allograft function in 1%–15%.

### Sources and Risks of BKPyV

The primary sources of BKPyV in kidney transplantation include environmental exposure [50], viral reactivation in the recipient [51], and donor-derived infections (DDI) [52]. Donor-derived BKPyV plays a significant role, with genomic sequencing often matching the donor’s BKPyV genotype. Recipients of kidneys from donors with urinary BKPyV shedding are at higher risk of viral replication [53]. Also, high antibody levels in the donors as a marker of significant or recent exposure, low and serotype-mismatched antibodies in the recipients have been identified as a risk factor for BKPyV-DNAemia/nephropathy [54, 55].

### New Guidelines for the Management of BKPyV in Kidney Transplantation

Pr Hirsch described the consequences on the clinical practice through summarizing [56]. The Second International Consensus Guidelines on the Management of BK Polyomavirus in Kidney Transplantation [57].

Diagnosis: Routine monitoring of plasma BKPyV-DNA levels up to 2 years post-transplant (3 years for pediatric patients). New-onset plasma BKPyV-DNAemia of >1,000 copies/mL sustained for 3 weeks was defined as probable BKPyV-nephropathy, plasma BKPyV-DNAemia of >10,000 copies/mL as presumptive BKPyV-nephropathy, and biopsy-proven BKPyV-nephropathy without and with impaired renal function, respectively. Biopsies should include systematic BKPyV-DNAemia assessment.

Management: Gradual reduction of immunosuppressants (e.g., mycophenolate, then tacrolimus) is recommended. Antiviral treatments like leflunomide and cidofovir lack strong clinical evidence. Retransplantation is viable if BKPyV-DNAemia clears, while allograft nephrectomy is deferred unless nephropathy persists.

### Future Directions

Further randomized trials are required to validate molecular diagnostics and safe antiviral strategies, including multi-virus-specific T-cell therapy, which shows promise for treating BKPyV nephropathy [58].

## The Donor With a Known and Treatable Infection: What Should We do?

J-R Zahar, Avicenne University Hospital, Paris, France did an interactive presentation about practical cases of donors with a known and treatable infection.

The shortage of transplantable organs highlights the need to correctly identify situations of potential organ availability. In case of an ongoing infection at the time of donation, there are a number of data available from different scientific societies [59, 60]. Simultaneous bacterial infections are often “unrecognized” at the time of transplantation and the diffusion of multidrug resistant micro-organisms is an additional risk for recipients. However, most case series acknowledge that bacterial infections transmitted by the donor are rare [61]. The risk of transmission depends on several factors such as the site of infection, the species involved, their antibiotic susceptibility profile, previous antibiotic treatment regimen and duration before and upon transplantation.

Four factors are to be considered (apart from immunosuppression treatment):

• The site of infection: the risk of donor-derived infection (DDI) seems to be different in the event of *in situ* infection, or at distance from the transplanted organ. The bacterial inoculum effect influences bacterial clearance, as high inoculum are associated with lower clearance, exemplified by intravascular infections in which bacterial DNA load are elevated [60].
• The microbial species: *Staphylococcus aureus*, *Klebsiella pneumoniae* and *Pseudomonas aeruginosa* are known to have a higher risk of morbidity and mortality in acute severe settings. Consequently, causal microbial species must be taken into account for treatment duration.
• The mechanisms of multidrug resistance and types of antibiotic treatment received: the growing spread of multidrug resistance across communities and hospitals expose transplant recipients to a higher risk of difficult-to-treat infections. Consistently, an assessment of the prior treatments received by the candidate recipient and the donor are essentials (bactericidal activity, dose, modalities of administration and duration) to guide any empiric antibiotic strategy upon transplantation procedure.
• The transplanted organ: some infectious risks are organ-specific. It is important to take into account the risk associated with the “non-drainable” nature of the infection and the capacity of the antibiotics administered to diffuse into the infected transplanted organ.

## Conclusion

During the well-attended “Infection and Transplantation Group” day, the major and recent advances in the field of the current risk of donor-transmitted infections in solid organ transplantation highlighted the crucial importance of vigilance and the constant updating of knowledge in this area. This led us to keep in mind key information:

- Detailed screening of potential organ donors is essential to detect infections that could be transmitted to recipients. This includes assessing medical history, previous infections, vaccinations, and exposures.
- Various infections pose risks, such as hepatitis viruses mostly HEV, BK virus, fungal infection
- Climate change involved the increase of emerging Infections. Diseases like West Nile Virus, Dengue, are particularly important in transplantation due to their potential transmission through organs, their increase severity-risk in those immunocompromised hosts in absence of specific treatment, and have been integrated in donor’s screening seasonally.
- The acceptance and outcomes of organ transplantation from HIV-positive donors to HIV-positive recipients are evolving, with successful cases.
- Globalization and travel contribute to the spread of infectious diseases, impacting donor screening protocols.

Overall, ensuring the safety of organ transplantation involves rigorous screening protocols, continuous monitoring, and adapting to emerging infectious disease risks. Each case requires careful consideration of risks versus benefits to optimize patient outcomes post-transplant, in the context of organ shortage.

## Footnotes

1
https://freepub.edqm.eu/publications
